# The Psychological and Biological Impact of “In-Person” vs. “Virtual” Choir Singing in Children and Adolescents: A Pilot Study Before and After the Acute Phase of the COVID-19 Outbreak in Austria

**DOI:** 10.3389/fpsyg.2021.773227

**Published:** 2022-01-04

**Authors:** Katarzyna Grebosz-Haring, Anna K. Schuchter-Wiegand, Anja C. Feneberg, Nadine Skoluda, Urs M. Nater, Sebastian Schütz, Leonhard Thun-Hohenstein

**Affiliations:** ^1^Department of Musicology and Dance Studies, Faculty of Art History, Musicology and Dance Studies, Paris Lodron University Salzburg, Salzburg, Austria; ^2^Programme Area (Inter)Mediation. Music – Mediation – Context, Interuniversity Institution Knowledge and the Arts, Paris Lodron University Salzburg, University Mozarteum Salzburg, Salzburg, Austria; ^3^Department of Clinical and Health Psychology, Faculty of Psychology, University of Vienna, Vienna, Austria; ^4^University Research Platform ‘The Stress of Life – Processes and Mechanisms Underlying Everyday Life Stress’, University of Vienna, Vienna, Austria; ^5^Department of Mathematics, Faculty of Natural Sciences, Paris Lodron University Salzburg, Salzburg, Austria; ^6^University Department of Child and Adolescent Psychiatry, Christian-Doppler-Clinik, Paracelsus Medical University, Salzburg, Austria

**Keywords:** virtual choral singing, in-person choral singing, music, children, adolescents, cortisol, COVID-19

## Abstract

Psychobiological responses to music have been examined previously in various naturalistic settings in adults. Choir singing seems to be associated with positive psychobiological outcomes in adults. However, evidence on the effectiveness of singing in children and adolescents is sparse. The COVID-19 outbreak is significantly affecting society now and in the future, including how individuals engage with music. The COVID-19 pandemic is occurring at a time when virtual participation in musical experiences such as singing in a virtual choir has become more prevalent. However, it remains unclear whether virtual singing leads to different responses in comparison with in-person singing. We evaluated the psychobiological effects of in-person choral singing (7 weeks, from January to March 2020, before the COVID-19 outbreak) in comparison with the effects of virtual choral singing (7 weeks, from May to July 2020, after schools partly re-opened in Austria) in a naturalistic pilot within-subject study. A group of children and young adolescents (*N* = 5, age range 10–13, female = 2) from a school in Salzburg, Austria were recruited to take part in the study. Subjective measures (momentary mood, stress) were taken pre- and post-singing sessions once a week. Additionally, salivary biomarkers (cortisol and alpha-amylase) and quantity of social contacts were assessed pre- and post-singing sessions every second week. Psychological stability, self-esteem, emotional competences, and chronic stress levels were measured at the beginning of in-person singing as well as at the beginning and the end of the virtual singing. We observed a positive impact on mood after both in-person and virtual singing. Over time, in-person singing showed a pre-post decrease in salivary cortisol, while virtual singing showed a moderate increase. Moreover, a greater reduction in stress, positive change in calmness, and higher values of social contacts could be observed for the in-person setting compared to the virtual one. In addition, we observed positive changes in psychological stability, maladaptive emotional competences, chronic stress levels, hair cortisol, self-contingency and quality of life. Our preliminary findings suggest that group singing may provide benefits for children and adolescents. In-person singing in particular seems to have a stronger psychobiological effect.

## Introduction

The need for cultural participation has been recorded since prehistoric times (Fancourt, 2017: 3). Recent research supports the hypothesis that music-related activities such as singing promise a wealth of positive applications to human behavior, health, and psychological well-being in both non-clinical and clinical populations, including the elderly, adults, children and adolescents (cf. MacDonald et al., 2012; Bernatzky and Kreutz, 2015; Kreutz and Nater, 2017; Grebosz-Haring and Thun-Hohenstein, 2018, 2020).

This corresponds to the evidence that music-related activities are capable of inducing emotional-affective phenomena and can generate and regulate emotions (Panksepp and Bernatzky, 2002; Juslin and Västfjäll, 2008; Koelsch, 2014), improve mood (Koelsch et al., 2010; Grebosz-Haring and Thun-Hohenstein, 2018), relieve stress (Pelletier, 2004; Thoma et al., 2012; Linnemann et al., 2015, 2017), and encourage social behaviors (Koelsch, 2013).

Moreover, neuroimaging studies of healthy participants have shown that the experience of music leads to the activation of manifold cortical and subcortical neural networks (Altenmüller and Schlaug, 2012) and the midbrain area that are implicated in emotions, reward, and motivation (Blood and Zatorre, 2001; Panksepp and Bernatzky, 2002; Bernatzky et al., 2015). Thus, there is emerging evidence that music experiences affect not only psychological mood changes but also induce biological responses (Grebosz-Haring and Thun-Hohenstein, 2018; for the possible effects of music-driven emotions on changes in hormone systems effectors, see Koelsch, 2014). The positive valence (pleasurable experience) of music seems to be of great relevance for these effects (see e.g., Blood and Zatorre, 2001; Brown et al., 2004).

Different biological responses to musical activities have been reported with respect to a range of biological outcomes such as salivary cortisol (e.g., Beck et al., 2000; Fancourt et al., 2016; Schladt et al., 2017) and salivary alpha-amylase (Nater et al., 2006) in healthy and clinical individuals (Koelsch and Stegemann, 2012; reviewed in Chanda and Levitin, 2013; Fancourt et al., 2014). Cortisol itself is a central active agent in the stress response, indicating activity of the hypothalamic-pituitary adrenal (HPA) axis (Kirschbaum and Hellhammer, 1994). Over the course of the day, the highest level is reached in the 30-45 min after waking up, the lowest is reached around midnight, whereas salivary alpha-amylase, indicating autonomic nervous system activity (ANS) (Rohleder and Nater, 2009; Strahler et al., 2017), shows the opposite pattern. The early afternoon can be considered a suitable time window for assessing potential changes in the activity of the HPA axis and the ANS induced for example by music-related activities (Berg et al., 2018). In contrast to these salivary biomarkers which reflect rather short-term and momentary fluctuations of the activity of the biological stress systems, cortisol accumulated in hair is considered a valid long-term marker of cortisol secretion over prolonged time periods (Stalder and Kirschbaum, 2012).

Choir singing in particular appears to be associated with positive biological response patterns in adults (Beck et al., 2000; Kreutz et al., 2004; Fancourt et al., 2016; Schladt et al., 2017). However, little is known about whether singing has a beneficial effect also in children and adolescents. In this context, preliminary studies of young people suggest that singing together in a group has a positive impact on psychological as well as biological indicators in this population (Grebosz-Haring and Thun-Hohenstein, 2018, 2020; overview in Glew et al., 2020). For example, adolescent patients with mental disorders who took part in a 5-day group singing intervention experienced a significant decrease in salivary cortisol levels compared to those in a 5-day music listening intervention (Grebosz-Haring and Thun-Hohenstein, 2018). Moreover, participation in a *Singing Medicine Project* helped children to express themselves and was associated with a reduction in negative emotions (Blackburn, 2020).

Studies in adults also suggest that group singing has a positive impact on social and emotional outcomes (Bullack et al., 2018; Moss et al., 2018), and can evoke a feeling of belonging and resilience (Daykin et al., 2020).

The COVID-19 outbreak and the stay-at-home and quarantine orders issued by governments produced the largest enforced isolation period in human history (Fancourt et al., 2020), which led to a radical change in people’s behavior and has had a serious impact on life for the majority of people all over the world, including children and adolescents. In Austria, this population had to stay home during lockdown in spring 2020 (from March 16th until May 15th) and were confined to home-schooling *via* internet. Interactive courses were offered, but it was not possible to have any real social meetings, such as visits with friends, or any sports or music education. About 10-15% of all children stopped attending school because of missing financial or technical support. Furthermore, unemployment levels rose and home office models increased significantly, thus increasing the pressure for families to adjust their lives during this and the following periods of pandemic-related lockdown. After the end of the lockdown, students in elementary, middle and high school programs returned to in-person classes. Classes were divided into smaller groups and taught on a rotating basis. In addition, there were strict hygiene measures in place and students were required to wear a mask whenever they left their assigned seat. Sports and music-related activities (in school or as an extracurricular activity) were not permitted anywhere.

The severe effect of the pandemic on children and adolescents has been documented in several areas (e.g., Ravens-Sieberer et al., 2020, 2021; de Figueiredo et al., 2021). For example, an online survey (Schabus and Eigl, 2021) of the emotional state of children and adolescents aged 6–18 years during the coronavirus pandemic in Austria shows that children and adolescents face significant challenges due to the situation and have difficulty seeing things in context. Furthermore, feelings of anger, annoyance, loneliness and sadness increase and there is an alarming deterioration in sleep quality and an increase in sleep problems (ibid.). The data from the study suggest quick action to curb the psychosocial, developmental and health-related damage in this young and vulnerable age group (ibid.). In addition, as children enter the pre-pubertal and pubertal age, profound changes take place in their socioemotional development, social behavior and status, and the functioning of stress-responsive systems. Interference in these adaptive developmental processes might increase the risk of behavioral problems and psychopathology (Spear, 2000; Foley and Weinraub, 2017; Roberts and Lopez-Duran, 2019; Jones et al., 2020). Therefore, particularly in times of lockdown and school cancelation, there is a need for easily available early interventions and prevention strategies in young individuals. In this context, musical activities such as choir singing seem particularly promising.

Music has played a great role in the Covid-19 pandemic. For example, data from a thousand individuals in Italy, Spain and the United States (Mas-Herrero et al., 2020) shows that music-related activities were the most popular ideas to cope with the psychological distress of the pandemic. Participants who were more severely affected by the COVID-19 pandemic reported a higher level of music-related activities during the first lockdown, which was a positive means of coping with psychological stress caused by the pandemic. The study also found that music-related activities were associated with decreased mental health symptoms during the pandemic, most likely mediated by the activation of pleasure and reward processes. Moreover, the study by Granot et al. (2021) found that music helps more than other daily activities in dealing with the crisis. It is an efficient means for achieving goals related to well-being under extremely stressful situations in different age groups, cultures and genders. Furthermore, it was suggested that music during the COVID-19 pandemic can regulate mood (Mas-Herrero et al., 2020; Cabedo-Mas et al., 2021) and stress (Fink et al., 2021), and reduces loneliness (Martiìn et al., 2021).

The study by Chiu (2020) compares the musical activities of the Milanese during an outbreak of the plague in 1,567 with the musical activities (e.g., balcony singing) during the Covid-19 lockdown and investigates how music regulates the mood and maintains social cohesion in such times. The Milanese in 1,567 used community music activities to cope with their condition, just as people in the last year met on their balconies and sang together to lessen their fears and isolation. People experienced a feeling of connectedness and happiness through the balcony singing. Furthermore, there are countless COVID-19 and quarantine-related playlists on music streaming platforms like Spotify that are created and shared by users. The social function of allowing people to connect through shared music made these playlists very meaningful for many people.

Virtual meetings during the lockdown have become popular to maintain social and professional life, including engagement with musical activities. Virtual choirs in particular provided a semblance of “normality” to many singers during the COVID-19 lockdown in the United Kingdom (Daffern et al., 2021). Participants realized through the loss of singing together how important choir singing was for their well-being. Virtual singing was a poor substitute, but since it maintains social contacts and a sense of well-being, it was better to have this opportunity than not to sing at all. People who are not so familiar with the internet encountered difficulties, as getting online can be challenging. In general, however, technology (e.g., better internet connections) must improve so that virtual choirs can become a part of our reality (ibid.). It is important to mention that different models of virtual singing were adopted, e.g., multi-tracked virtual choir (see e.g., Fancourt and Steptoe, 2019), which is not real-time virtual singing and different from in-person choir singing. Theorell et al. (2020) examined what happens when choir singers lose their routines due to COVID-19. The authors found that the social aspect of singing was perceived as the greatest loss.

However, the effectiveness of virtual choir singing in young people during the COVID-19 lockdown requires further investigation. This is important because understanding the patterns of singing across lockdowns could help organizations plan for future musical activities. Furthermore, understanding how young people respond to virtual singing could enhance our understanding of the effect of this art of musical activity on mental health and well-being.

Virtual singing has become prevalent as a helpful method to connect with other people. Virtual musical activities may remain important to the well-being of individuals who cannot participate in in-person musical activities (Fancourt and Steptoe, 2019). A study on virtual singing in adults (Fancourt and Steptoe, 2019) examined the differences in the perception of social presence and the use of emotion regulation strategies between singers of an in-person choir and singers of a virtual choir. According to this study, singing in a virtual choir was associated with a higher overall perception of social presence, as the singers used more self-development strategies, fewer emotion-regulation strategies and fewer avoidance strategies (for example distraction) than singers in an in-person choir. Possible explanations could be that members of a virtual choir are treated both as group members and as soloists at the same time; they could improve themselves by recording their contributions; and the decision to sing in a virtual choir could go along with the desire to improve self-confidence in singing (ibid.).

However, it remains unclear whether a virtual musical activity such as choir singing is feasible in children and adolescents and whether it leads to the same psychobiological responses as in-person singing in this group of participants. Consequently, the present study aims to explore the potential psychobiological effectiveness of in-person and virtual choir singing on children and adolescents and to assess whether virtual singing has the same effects as in-person singing during a strict lockdown period.

## Materials and Methods

### Study Design and Participants

The data from this naturalistic pilot within-subject study of in-person group singing versus virtual group singing were collected in the context of a control trial registered at ClinicalTrials.gov (Identifier: NCT01921088^1^).

The goal of this larger study was to examine the biopsychological responses to choir singing (amateur and professional) in different clinical and healthy populations of children and adolescents and to understand the biopsychological mechanisms underlying choral singing in order to determine its full potential, particularly for vulnerable populations. The study protocol was approved by the Salzburg State Ethics Committee (reference number 415-E/2554/5-2019).

The whole study consisted of five months of singing interventions and included children and adolescents (10–18 years) with mental disorders, healthy children and adolescents from schools in Salzburg, and members of the Vienna Boys’ Choir in Austria. The study commenced on January 16*^th^*, 2020 at the Christian Doppler Gymnasium in Salzburg, and on January 13th at the Upper Secondary School of the Vienna Boys’ Choir. Recruitment at the schools lasted from September 2019 until January 2020. On March 6th, 2020, as a result of the first strict lockdown (stay-at-home order) because of the COVID-19 pandemic, the choir sessions had to be stopped; in order to continue the project after the first acute phase of COVID-19, the study was adjusted and the in-person singing activity at the Christian Doppler Gymnasium in Salzburg was continued as a virtual singing activity. The choir sessions at the school in Vienna had to be stopped.

**FIGURE 1:**
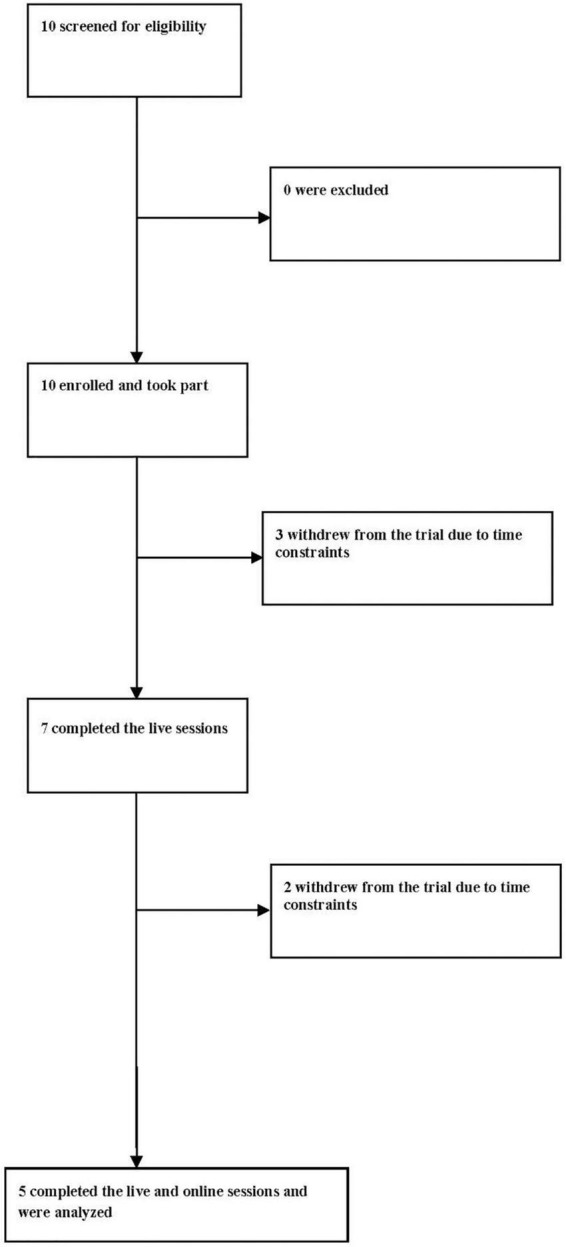
Study flow.

For the current analysis, we used data from one of the researched groups, namely, from healthy students at the Christian Doppler Gymnasium in Salzburg who participated in the in-person choir for the first few weeks before the COVID-19 lockdown period and later on participated in the virtual choir singing intervention that was continued after schools partly re-opened as the lockdown was eased. 10 participants were screened for eligibility to participate in a choir and 10 were enrolled. All 10 enrolled patients entered the singing sessions. Three participants dropped out of the in-person singing sessions and two dropped out of the virtual singing sessions because of difficulties with time management. In the statistical analysis, we only included participants who took part in both the in-person and virtual singing sessions. Overall, 5 (50%) participants completed the study and were analyzed (see Figure 1 for study flow).

The inclusion criteria were that participants had to be 10–18 years old. Participants were ineligible if they were diagnosed with any significant hearing impairments (according to a self-report and the patient’s file) or an inability to verbalize. Participation in the study was voluntary. Written informed consent from each participant and parent or legal guardian was obtained prior to the study. Compensation of two Euros per choir session and of three Euros for completing the data at three additional time points (at the beginning of in-person singing as well as at the beginning and the end of the virtual singing) was given to every participant. The changes to the pilot study (e.g., moving virtual) were approved by the Salzburg State Ethics Committee.

### Procedure

We wanted to maintain the original study design as much as possible and therefore did this pilot study according to our definitive controlled study.

Participants took part in seven in-person singing sessions running from January 16th to March 5th 2020 (7 weeks before the COVID-19 outbreak). The in-person singing sessions were provided as a 45-min group session once a week. Due to circadian variations in salivary biomarkers, sessions were carried out in the afternoon between 1:00 and 4:00pm. Prior to the first choir singing session, parents delivered data regarding socio-demographic characteristics. The participants also filled out a questionnaire on musical background. Additionally, they completed a series of questionnaires (for details, see Chapter 2.3. Data) and provided hair samples. Baseline data was filled out by students with the help of a research assistant at their school.

Subjective measures of participants’ momentary mood and stress were administered pre- and post singing session. Additionally, the participants gave feedback regarding their aesthetic experiences of the choir activity after each session. Salivary biomarkers and social contacts were assessed pre- and post singing sessions every second week. When possible, participants were asked not to consume any meals, alcoholic drinks, coffee, tea, cola, juices, or chewing gum, and to refrain from smoking for one hour before the measurement of each singing session.

All sessions were led by a professional choirmaster. Each choir session initially focused on an approximately ten-minute-long physical activation, vocal warm-up (sounding, making the voice sound, coming out of the speaking voice, increasing high and low resonance), and attention exercises. Throughout the rest of the session, new songs in various styles and languages chosen by the choirmaster as well as songs familiar to the participants were rehearsed (see Appendix 1 for the used songs literature and Appendix 2 for a detailed description of the choir session). Due to the practical considerations in a rehearsal choir situation, the singing repertoire was chosen by the choral conductor; however, there was also a possibility for participants to choose their own songs, including familiar ones. During the singing sessions, the choral conductor frequently asked the participants for feedback on the songs. He asked them if they liked them and if they wanted to sing the songs again. He was open to any new repertoire and allowed the choir members to participate in the decision-making process. In selecting repertoire, the conductor prioritized pieces that were not too difficult for casual singers and that were also liked by the young participants. Previous studies indicated that participants’ familiarity with and liking of songs had a positive effect on their cortisol levels (Grebosz-Haring and Thun-Hohenstein, 2018) and led to the activation of specific limbic and reward-processing areas of the brain that regulate endocrine responses to favored, or self-chosen (Blood and Zatorre, 2001; Salimpoor et al., 2011), and pleasurable music (Brown et al., 2004). We therefore assume that these conditions in the singing sessions may have contributed to the positive psychobiological effects. Furthermore, the chosen repertoire was intended to involve different kinds of vocal work, including musicality, diction, timbre and part-singing.

After schools partly re-opened in Austria, the participants took part in seven virtual singing sessions running from May 28th to July 9th 2020. The duration of the virtual choir singing sessions and the procedure were the same as in the in-person choir singing sessions.

For the virtual sessions, participants were asked to meet the research assistant in front of their school in different time slots to receive the score sheets for the choir sessions, the series of questionnaires, materials for saliva sampling, and the equipment for the hair samples. The material was handed over to each person in an envelope so an appropriate distance could be maintained. Subjects completed the same series of questionnaires and provided hair samples at the beginning and at the end of the virtual singing sessions. Due to the COVID-19 situation, participants had to fill out the survey at home on their own. Participants were offered online help from the research assistant whenever needed. The within-session (pre-post singing session) measurements were conducted online (in a digital format) with the help of the research assistant.

Virtual singing sessions took place through the video-conferencing platform Zoom (Zoom Video Communications, San José, California). The participants sang the same kinds of songs as in the in-person singing sessions (see Appendix 1). New songs were introduced regularly to expand the repertoire (see Appendix 1). In the virtual choir, participants could see each other and sang together in real-time. However, participants were in their own individual physical locations and could only hear the choirmaster and themselves singing solo; they were not able to hear other choir members, who sang in parallel over the video call. Due to the technical limitations of Zoom, the choirmaster was also unable to hear the choir members. Sometimes one or two participants had connection problems with the internet and were unable to adequately participate in the choir session. They logged in and out repeatedly, but other participants continued to sing without interruption. Some had switched off their cameras, but turned them on again when the choir conductor explicitly requested that all cameras should be switched on so that everyone could see each other and feel more like they were part of a choir.

### Data

We collected the data on a number of outcome measures used in a definitive study, which would allow us to address our research questions and to assess the acceptability and usefulness of the measures for inclusion in a larger study as well.

#### Socio-Demographic Variables and Musical Background

At baseline, parents filled out questionnaires on socio-demographic variables (see Table 1 for the used variables). Additionally, participants filled out a questionnaire on their habitual music preferences (MPQ-KJ; adapted version for children and adolescents based on the adult version Music Preference Questionnaire-revised; MPQ-R; Nater, 2003). Musical background (*current and past musical activities*) was assessed through dichotomous *yes* vs. *no* items *(I play/have played one or more instruments, I take/took singing lessons, I am playing/was playing in a band, I am singing/was singing in a choir, I am/was a member of an orchestra*).

**TABLE 1:** Participant characteristics.

		(*n* = 5)	ID 2	ID 3	ID 4	ID 5	ID 7
Age (years): median		10	10	10	13	13	10
Age max		10					
Age min.		10					
**Gender**							
	Female	2	1	0	0	0	1
	Male	3	0	1	1	1	0
**Citizenship**							
	Austria	5	1	1	1	1	1
**Parent income (net)**							
	1,000–1,999	1	0	0	0	1	0
	2,000–2,999	1	1	0	0	0	0
	3,000–3,999	1	0	0	1	0	0
	4,000–4,999	1	0	0	0	0	1
	5,000–5,999	1	0	1	0	0	0
**Finance comparison to general population**							
	slightly worse	1	0	0	0	1	0
	slightly better	3	1	0	1	0	1
	markedly better	1	0	1	0	0	0
**Employment status mother**							
	Employed	3	0	1	1	0	1
	Unemployed	1	1	0	0	0	0
	In education	1	0	0	0	1	0
**Employment status father**							
	Employed	4	1	1	1	0	1
	Not declared	1	0	0	0	1	0
**Education mother**							
	Compulsory school	1	1	0	0	0	0
	Higher School Certificate	2	0	0	1	0	1
	University degree	2	0	1	0	1	0
**Education father**							
	Vocational school	1	0	0	0	1	0
	Higher School Certificate	3	1	1	0	0	1
	University degree	1	0	0	1	0	0
**Musical background**							
	Currently playing an instrument	2	0	0	1	0	1
	Currently singing in a choir	1	0	1	0	0	0
	Previously playing an instrument	3	0	1	1	0	1
	Previously singing in a choir	1	1	0	0	0	0
	Previously playing in an orchestra	1	0	0	1	0	0
**Mental health diagnosis**							
	No	4	1	0	1	1	1
	Not declared	1	0	1	0	0	0
**Other medical diagnosis**							
	Yes	1	0	0	1	0	0
	No	3	1	0	0	1	1
	Not declared	1	0	1	0	0	0

#### Perception of Singing Sessions

The participant self-report questions on aesthetic experience were used to assess the perception of the singing session. Participants were required to rate how much they liked the choir session (*How much did you like singing in the choir today?)* and how much they liked the songs (*Did you like the songs?*) as well as their familiarity with the songs (*Did you know the songs?*) on a 5-point Likert scale ranging from 1 (*not at all* or *no*) to 5 (*very much* or *yes, all*) immediately after every choir session. High values are indicative for high liking and familiarity with the songs.

#### Subjective Stress Experience

*Current subjective stress* was assessed using a “Visual Analogue Scale” (VAS) single-item approach as suggested by Elo et al. (2003). Participants rated their momentary stress level (*How stressed do you feel at the moment?*) between 0 (*not at all*) and 100 (*very much*). A higher score indicates a higher level of subjective stress (Linnemann et al., 2017).

#### Subjective Momentary Mood

*Current mood state* was assessed using the “Multidimensional Mood Questionnaire” (MDMQ; Steyer et al., 1997; short form A), which consists of three bipolar scales: *good - bad mood* (GM), *alertness - tiredness* (AT), and *calmness - restlessness* (CN). The MDMQ is a well-validated tool for screening current mood state in clinical practice and research and is specifically appropriate for repeated measures within short intervals, with 12 items (4 items - 2 positive and 2 negative - on each of the three scales) on a five-point rating scale. For every subscale, the values of the corresponding items are added up, ranging from 4 to 24 per scale. A higher score suggests positive affectivity, wakefulness, and calmness, respectively.

#### Social Contacts

We assessed *quantity of social contacts* within the choir using the “Social Network Map” adapted to application with children and adolescents (Tracy and Whittaker, 1990; see also Linnemann et al., 2017) every second week (1, 3, 5, 7 in-person singing sessions and 9, 11, 13 virtual singing sessions) post singing sessions. The Social Network Map uses a graphical representation (so-called circle mapping technique; Tracy and Whittaker, 1990) to provide information on the network size of each participant within the choir. Every participant rated familiarity with the other participants, classified as best friends, friends, or acquaintances. Participants were asked to write down a number or symbol within the classifications for privacy reasons (Linnemann et al., 2017). The analysis of the social network map is based on the overall number of identifications, as an indicator of the quantity of social contacts within the choir (Tracy and Whittaker, 1990). A higher total number for the network indicates a higher social community within the choir (Linnemann et al., 2017).

#### Biological Measurements of Momentary Stress

Saliva samples for the analysis of salivary cortisol (sCort) as an indicator of the HPA axis as well as salivary alpha-amylase (sAA) as a marker indicating ANS activity were collected directly before and after every second choir session (1, 3, 5, 7 in-person singing sessions and 9, 11, 13 virtual singing sessions). Changes in values of sCort and sAA indicate ultimate responses of both stress-responsive systems to external situations and have been used in studies investigating the biopsychological effects of choir singing in previous studies (e.g., Kreutz et al., 2004). Before the sample collection, participants were instructed to rinse their mouths with water and to accumulate unstimulated saliva in the oral cavity for two minutes. A research assistant indicated when two minutes had passed and signaled the participants to transfer the saliva into polypropylene tubes *via* a straw (SaliCap^®^, IBL, Hamburg, Germany).

During the virtual choir sessions, participants were asked to conduct the procedure for the collection of saliva at home before and after every second session. They were always instructed *via* video using the Zoom platform. Saliva samples were cooled in the freezer at home for a few days before a research assistant collected them on the next school day.

The saliva samples were stored in a freezer at −20°C at the Central Laboratory of the Christian-Doppler-Clinic in Salzburg until they were sent on dry ice for cooling purposes to the Biochemical Laboratory of the University of Vienna for analysis. Concentrations of sCort were measured using a commercially available luminescence immunoassay (IBL, Hamburg, Germany). sAA activity was determined from saliva samples using an enzymatic photometric test from DiaSys (DiaSys Diagnostics, Holzheim, Germany). sCort is reported in nmol/l and sAA activity is reported in U/ml. Intra- and interassay variances for sCort and sAA were below 10%.

#### Psychological Measurements of Psychological Stability, Self-Esteem, Emotion Regulation Strategies, Chronic Stress, and Quality of Life

These data were obtained at three time points in total: at the beginning of the in-person choir singing intervention and at the beginning and the end of the virtual choir singing intervention.

*Psychological stability* (parents’ view) was assessed using the “Child Behavior Checklist” (CBCL; Döpfner et al., 2014). This checklist consists of problem scales which describe behavioral problems, emotional problems and physical problems. The total score of the scale was analyzed. A higher total score reflects less stability.

*Self-esteem* was assessed with the “Self-Esteem Inventory for Children and Adolescents” (SEKJ; Schöne and Stiensmeier-Pelster, 2016). The SEKJ consists of three scales with 23 items overall: Self-esteem level (H), Self-esteem stability (S), and Self-esteem contingency (K). A higher score on each scale suggests a positive self-esteem level.

*Emotion regulation strategies* were assessed using the “Instrument to Measure Emotion Regulation Strategies in Children” (FEEL-KJ; Grob and Smolenski, 2009). Adaptive and maladaptive strategies were analyzed using sum scores. A higher score in adaptive strategies means higher use of the strategy in regulating feelings, which is positive for well-being. A higher score in maladaptive strategies means higher use of the maladaptive strategy, which is detrimental to well-being.

*Subjective chronic stress* was assessed using an adapted version of the “Chronic Stress in Childhood Questionnaire” (CSiK; Richartz et al., 2009). The CSiK consists of 12 subscales with 41 items overall: *school overload/pressure to perform, worries/social overload, social pressure, discontent with school, social tension, social isolation in the family, conflicts with siblings, temporal overload, social isolation among peers*. The higher the score of each subscale, the higher the level of chronic stress. The CSiK was originally developed for children between 8 and 10 years of age based on the Trier Social Stress Scale (TICS; Schultz et al., 2004) for adults. Since perception of stress changes as children transition from childhood to early adolescence and beyond (Seiffge-Krenke et al., 2009), we decided to slightly adapt certain items in consultation with the authors of the CSiK.

We assessed *quality of life* using the Pediatric Quality of Life Inventory (PedsQL; Varni et al., 2001). Quality of life is measured with 23 items using a 5-point Lokert scale ranging from 0 (*Never*) to 4 (*Almost always*). Each item is categorized under one of the 4 subscales: *Physical Functioning* (8 items), *Emotional Functioning* (5 items), *Social Functioning* (5 items), and *School Functioning* (5 items). The subscales are summarized into the *Psychosocial Health Summary Score* (= sum of the items over the number of items answered in the Emotional, Social, and School Functioning Scales) and into the *Physical Health Summary Score* (= Physical Functioning Scale Score). The *Total Score* is based on the sum of all four scales. The higher the score, the better the quality of life.

#### Long-Term Markers of Cortisol Secretion

Hair cortisol was collected as a long-term indicator of cortisol secretion (Stalder and Kirschbaum, 2012). For the assessment of hair cortisol, 2-3 thin hair strands were collected close to the scalp in the posterior vertex region. For the baseline assessment, the samples were taken on site by a research assistant. For the intermediate and final survey, due to the Covid-19 situation and to avoid risk of contagion, participants were asked to take the samples at home with the help of their parents, and to bring them to a prearranged meeting point in front of their school. Cortisol accumulates in human hair and hair has a growth rate of about 1 cm per month (Wennig, 2000). Thus, 1 cm of hair close to the scalp is indicative of chronic stress during the past month. In the present study, we used 1.5 cm segments, since this length covered almost the whole time frame between the intermediate and the final assessment. Each hair segment was washed twice with 3 mL isopropanol to remove external contaminants and, after drying, was finely cut into 1-2 mm pieces; 10.0 ± 0.5mg of finely cut hair was then transferred to a 20 mL glass tube and 1.8 mL methanol was added to extract hair cortisol overnight for 18 h. Afterward, 1.6 mL of the extract methanol supernatant was transferred to a 3 mL glass tube and subsequently evaporated at 50°C under a stream of nitrogen until it was completely dry. For the determination of cortisol, samples were resuspended with 225 μL of ultra-pure water and then analyzed using luminescence immunoassay (IBL, Hamburg, Germany). Hair cortisol concentrations (HCC) are reported in pg/mg.

### Statistical Analysis

First, data was analyzed descriptively. Due to the small sample size and ordinal scale of the majority of the analyzed variables, the median along with the minimum/maximum respectively interquartile range (IQR) were calculated. Graphical representation was achieved with boxplots, scatter plots and line plots.

Due to the small sample size and the ordinal scale of the data, non-parametric ANOVA-type statistics were used to compare longitudinal data, and the corresponding effect measure (Relative Treatment Effect [RTE]) was calculated according to Noguchi et al. (2012). Calculating the RTE is useful in studies with a small sample size or scores that are composed of several items. An RTE > 0.5 indicates a tendency toward increased scores, whereas an RTE < 0.5 indicates a tendency toward decreased scores at a certain time point relative to all timepoints. When presenting the results of inferential analysis, the comparison between pre and post sessions was always made first, regardless of the setting (virtual/in-person). Next, the effects of in-person and virtual settings were compared, followed by additional analyses of and observations in the data. No inferential analysis was performed for test formats with three timepoints of measurement. These data were presented in a descriptive way only. Due to the exploratory style of the analyses, no p-value correction was applied. All tests were carried out at the 5% significance level. All calculations were performed using the statistical software R, version 4.0.2, with the package nparLD (Noguchi et al., 2012).

## Results

### Participant Characteristics

Demographic characteristics of the participants are shown in Table 1. The median age of the five participants was 10 years, two participants were female and all participants were Austrian citizens. Most of the parents had a Higher School Certificate or above (mother and father *n* = 4) and were employed (mother *n* = 3; father *n* = 4). Household income varied among all participants from very low to very high; however, the majority of parents described their financial status as being better than the general population (*n* = 4). Participants reported having no diagnosed mental illness. Two of the participants were currently playing an instrument and one was currently singing in a choir. Three of the participants had played an instrument previously, one had been singing in a choir before and one had been a member of an orchestra earlier. None of the participants was taking one-on-one singing lessons or was a member of a band or an orchestra at the time of the study participation (see Table 1).

### Perception of Singing Sessions

**FIGURE 2:**
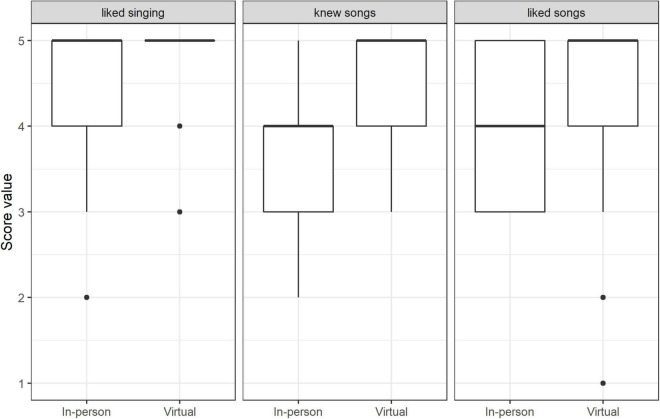
Score values of singing activity perception (median and range): in-person singing vs. virtual singing (*n* = 165 data points for all categories).

Participants liked both types of singing (in-person and virtual: median 5 on the 5-point Likert scale; see Table 2). The settings were rated equally by the participants in terms of the perception of singing sessions (*liking singing in today’s session*; Table 2), but the perception of the songs (*liking songs in today’s session*) in virtual singing setting was slightly higher than in the in-person setting (Figure 2). This resulted in a higher number of lower ratings (three and four on the five-point scale) in the in-person setting. Furthermore, in the virtual setting, the songs were more familiar to the participants than in the in-person setting (Table 2; see also Figure 2).

**TABLE 2:** Median and range for singing session perception by activity.

		Median (Range)
Liking of choir sessions	In-person	5 (2 - 5)
	Virtual	5 (3 - 5)
Familiarity with songs	In-person	4 (2 - 5)
	Virtual	5 (3 - 5)
Liking of song	In-person	4 (3 - 5)
	Virtual	5 (1 - 5)

### Subjective Stress Experience

**FIGURE 3:**
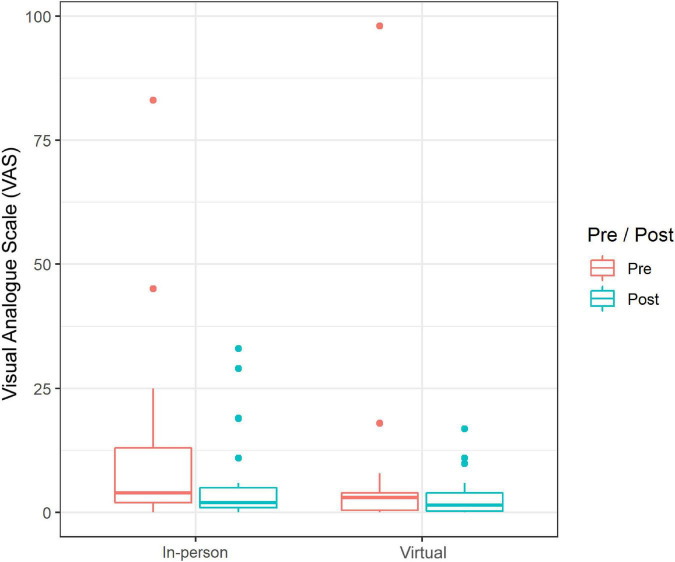
Absolute score values (pre-post) of current stress experience (median and range) on the Visual Analog Scale: in-person singing vs. virtual singing (*n* = 111 data points).

Subjective stress was reduced pre to post by means of RTE from 0.58 to 0.42 without distinguishing between the type of singing setting (in-person vs. virtual); however, this change was not significant (*p* = 0.27). A comparison of the two settings showed a tendency toward different effects (*p* = 0.09). It seemed that there was a greater reduction of VAS in the in-person setting compared to the virtual one (RTE in-person: 0.75 to 0.49 vs. RTE virtual: 0.41 to 0.36). However, we observed higher values in general at the beginning of the sessions in the in-person setting in combination with higher scattering (Figure 3). In general, the values in both settings were very low (positive) overall according to the pre-measurements.

### Subjective Momentary Mood

**FIGURE 4:**
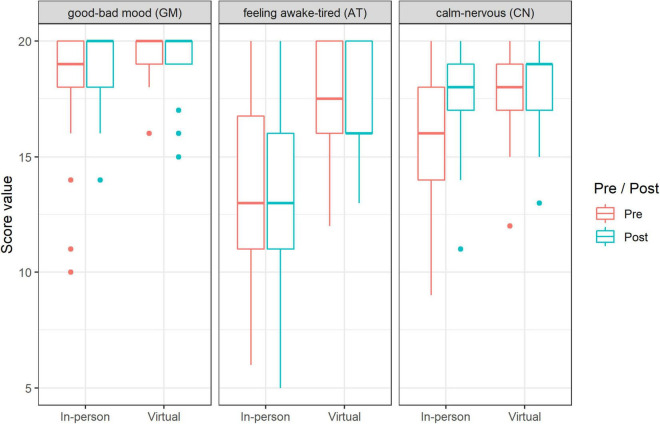
Changes (pre-post) in the MDMQ subscales (median and range): in-person singing vs. virtual singing (*n* = 330 data points).

We found marked changes in pre-post-values of the current mood state, in the dimension mood valence as an effect of singing (independent of singing setting). On the basis of RTE, it can be observed that overall, the pre-values were lower than the post-values (RTE: 0.45 to 0.54; *p* = 0.01; Figure 4). Furthermore, a comparison of both singing settings in MDMQ scales showed that there was a significant pre-post difference between the settings in current mood state in the dimension calmness (*p* = 0.001): in-person sessions brought an increase in the dimension calmness (RTE 0.3 to 0.56), while the value in the virtual setting remained almost the same (RTE 0.55 to 0.58; Figure 4). No significant pre-post differences between the settings were found in the dimensions mood valence and alertness. Interestingly, the values (both pre and post sessions) in the virtual setting were significantly higher in all of the three scales (mood valence *p* = 0.06; calmness *p* = 0.004; alertness *p* = 0.003) compared with the in-person setting (see Figure 4). However, in general, we observed that the values in the mood scales were very high (positive) at the beginning of sessions in both settings (in-person/virtual; see Figure 4).

### Social Contacts

**FIGURE 5:**
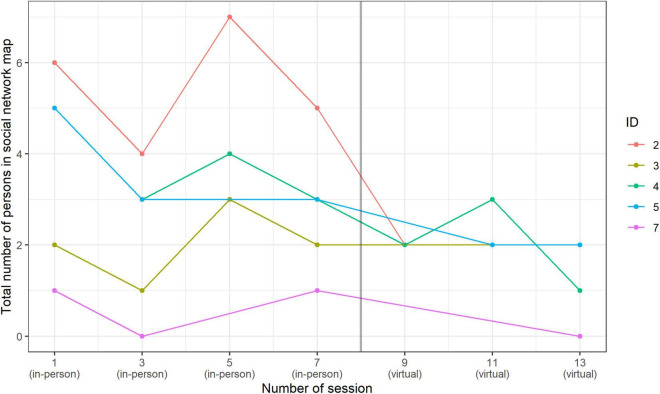
Number of social contacts in Social Network Map by session: in-person singing vs. virtual singing.

The social network map shows that the values for the in-person singing setting were higher than for the virtual singing setting; not unexpectedly, during the virtual singing setting, we observed a reduction in the number of social contacts (Figure 5).

### Salivary Cortisol

**FIGURE 6:**
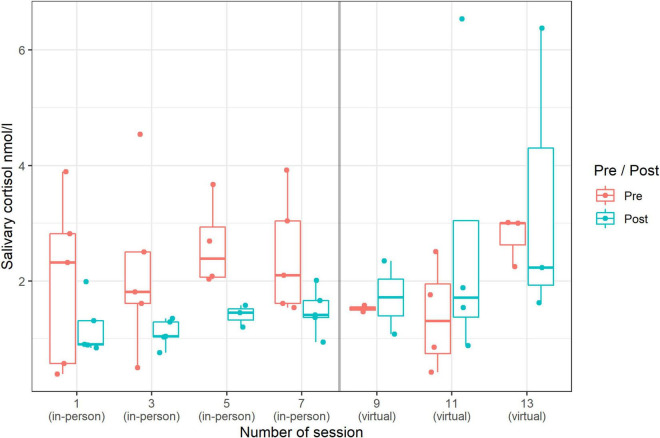
Changes (pre-post) in sCort (median and range) by session: in-person singing vs. virtual singing (*n* = 55 data points).

Overall, across all data, the results showed significantly lower sCort values after the singing sessions (independent of singing setting): the RTE pre-value was 0.59 and post-value was 0.40 (*p* < 0.001; Figure 6). Furthermore, a comparison of the pre to post sessions in the two settings (in-person/virtual) indicated a tendency toward different effects (*p* = 0.06). While a decrease of sCort pre to post session was observed in the in-person setting, in the virtual setting the level of sCort even increased pre to post session (RTE in-person: 0.70 to 0.22 vs. RTE virtual: 0.50 to 0.59). In general, we observed that over time, the level of sCort in the pre-values remained relatively the same in in-person sessions; the post-values even increased over the course of the samples, but there was still always a pre-post decrease (see Figure 6). In contrast, the progression in the virtual setting is somewhat variable. The median remained relatively the same in the first session, increased in the second session and decreased in the third session. However, it should be noted that very little data was available for the virtual sessions (e.g., in the second session, data was only available from two participants) and only three measurements (not four, as with the in-person sessions) took place (see Figure 6).

### Salivary Alpha-Amylase

**FIGURE 7:**
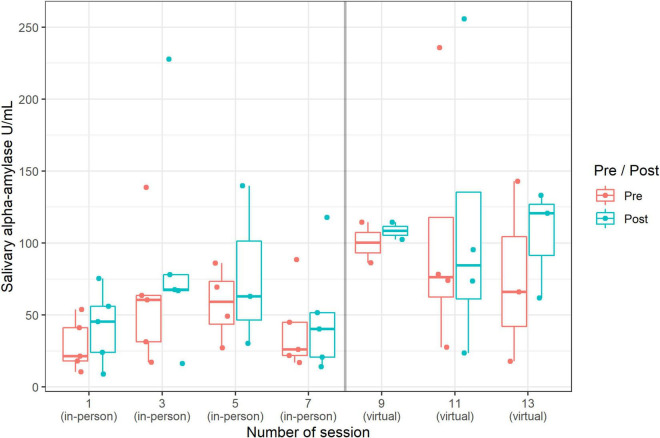
Changes (pre-post) in sAA (median and range) by session: in-person singing vs. virtual singing (*n* = 55 data points).

Overall, the data showed significantly higher values of sAA after the singing sessions (independent of in-person/virtual singing; RTE pre 0.46 to 0.54; *p* = 0.02). A comparison of the effect of the singing settings showed no significant differences in the pre-post-values of sAA. However, interestingly, we observed significantly higher values of sAA in general (regardless of pre to post) in the virtual singing setting compared to the in-person singing setting (RTE virtual: 0.67; RTE in-person: 0.33) (Figure 7).

### Psychological Stability (Parents’ View)

**FIGURE 8:**
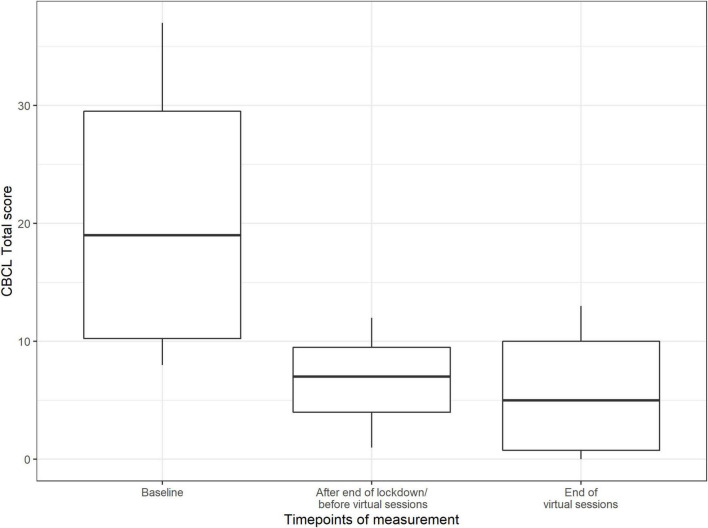
Score value on Child Behavior Checklist by timepoints of measurement (median and range) (*n* = 11 data points).

In Figure 8, the results are shown in a box-plot graph demonstrating a visible reduction of psychopathological symptoms over the three time points. The reduction occurred mainly between the first and second time points of the measurements. A slight decline in psychopathology was observed between the second and third times point of the measurements (see Figure 8).

### Self-Esteem

**FIGURE 9:**
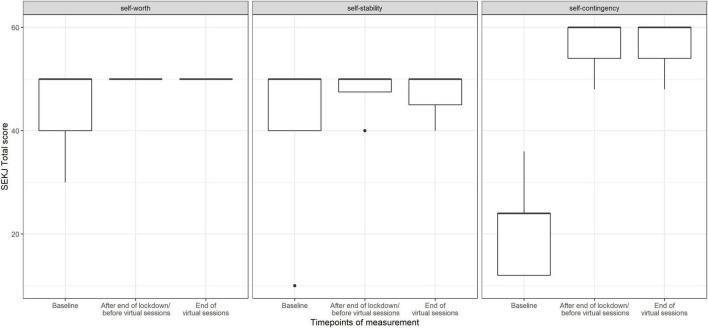
Score value on SEKJ scales by timepoints of measurement (median and range) (*n* = 35 data points).

Results for the SEKJ show no change in the subscales self-worth and self-stability, but a distinct increase in self-contingency at time point two, which remained high until the end of the study (see Figure 9).

### Emotion Regulation

**FIGURE 10:**
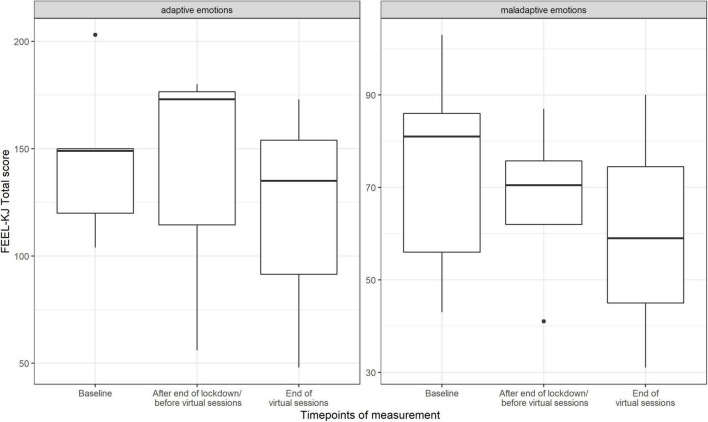
Score value on FEEL-KJ scales (adaptive emotions, maladaptive emotions) by timepoints of measurement (median and range) (*n* = 23 data points).

For results of FEEL-KJ, see box-plot graph in Figure 10. Between the first and second time point, the adaptive emotion regulation total score increased, and between the second and third time point, it went back to the values at the first time point. In contrast, the maladaptive emotion regulation total score decreased across the three time points, thus improving over time. According to the subscale levels, the median for adaptive regulation increased for adaptive anxiety and grief at time point 2 and returned to the values of period 1, but this did not apply to adaptive anger. Maladaptive anxiety and grief showed a continuous decrease over all three time points.

### Chronic Stress

**FIGURE 11:**
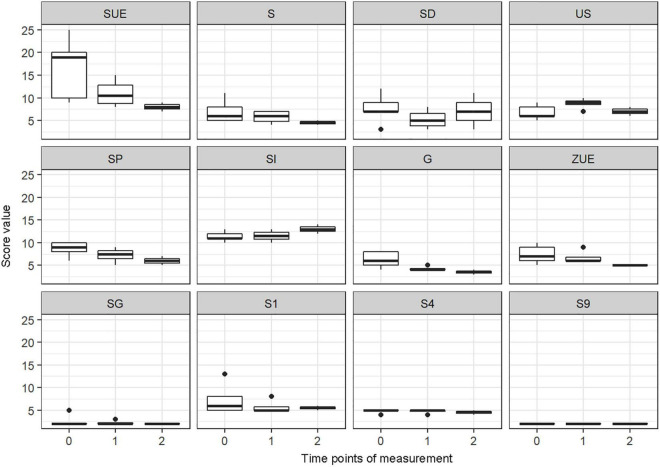
Score value on CSIK scales: school overload/pressure to perform (SUE), worries/social overload (S), social pressure (SD), discontent with school (US), social tension (SP), social isolation in the family (SI), conflicts with siblings (G), temporal overload (ZUE), social isolation among peers (SG), excessive demands/pressure to perform training/choir (S1), dissatisfaction with training/choir sessions (S4), social isolation in the training group/choir community (S9) by timepoints of measurement (median and range) (*n* = 132 data points).

Results of the CSIK show a continuous decrease in the values of the scales SUE (school overload/pressure to perform; Median baseline: 19; Median intermediate: 10.50; Median post: 8.0), S (worries/social overload; Median baseline: 6.0; Median intermediate: 6.00; Median post: 4.0), SP (social tension; Median baseline: 9.0; Median intermediate: 7.50; Median post: 6.0), G (conflicts with siblings; Median baseline: 6.0; Median intermediate: 4.00; Medina post: 3.5), and ZUE (temporal overload; Median baseline: 7.0; Median intermediate: 6.00; Median post: 5.0). The scale SD (social pressure) showed a decrease and an increase (Median baseline: 7.0; Median intermediate: 5.00; Median post: 7.0), whereas the scale US (discontent with school) showed an increase and decrease (Median baseline: 6.0; Median intermediate: 9.00; Median post: 7.0). The scale SI (social isolation in the family) showed an increase during the time period (Median baseline: 11.0; Median intermediate: 11.50; Median post: 13.0). Scales SG (social isolation among peers), S1 (excessive demands/pressure to perform training/choir), S4 (dissatisfaction with training/choir sessions) and S9 (social isolation in the training group/choir community) did not change during the time period (see Figure 11).

0 = Baseline

1 = After end of lockdown/before virtual sessions

2 = End of virtual sessions

### Quality of Life

**FIGURE 12:**
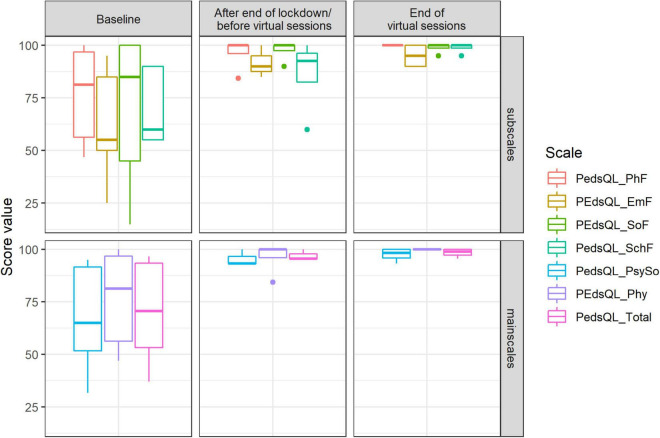
Score value on PedsQL scales: physical functioning (PhF), emotional functioning (EmF), social functioning (SoF), school functioning (SchF), psychosocial health summary score (PsySo), physical health summary score (Phy), total score (Total) by timepoints of measurement (median and range).

Figure 12 shows the results from the PedsQL. During the baseline measurement, the range of the subscales varied. The emotional functioning scale showed the lowest value compared to the other subscales, followed by school functioning, physical functioning, and social functioning. The values of every scale increased visibly during the period after the end of the lockdown, before the virtual sessions. After the end of the virtual choir singing intervention, values remained high. The total score, based on the sum of all four scales, increased after the in-person choir activity and remained high until the end of the virtual sessions.

### Hair Cortisol

**FIGURE 13:**
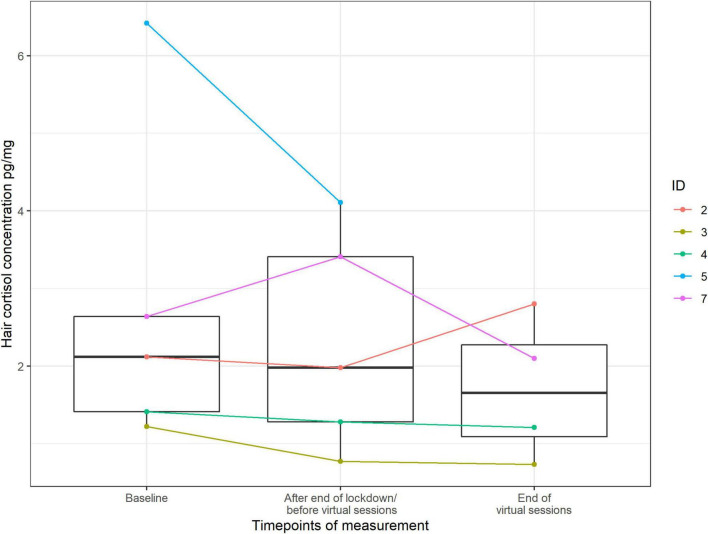
Score value of hair cortisol by timepoints of measurement (median and range) (*n* = 88 data points).

Hair cortisol concentrations seemed to decrease from the baseline (Median: 2.120) to the intermediate (Median:1.980) and from the intermediate to the final assessment (Median:1.655) (see Figure 13).

## Discussion

The aim of this naturalistic pilot study was to examine the psychological and biological effects of choir singing on children and adolescents and compare the psychobiological effects of in-person and virtual choir activities in children and adolescents before and after the acute phase of the COVID-19 outbreak and resulting lockdown in Austria. In carrying out this study, we adhered as much as possible to the parameters of our definitive larger controlled study. Our results indicate that singing – especially as an in-person activity – is likely to have potential psychobiological benefits for children and adolescents. However, the pilot design and small sample size mean that caution must be taken in interpreting the results. This is the first study to investigate this issue in this population.

Results show that in general, participants enjoyed singing. They liked both the in-person and the virtual singing sessions, but they liked songs more in the virtual setting. An explanation for this could be that the participants were more familiar with the songs and evaluated them more favorably than in the in-person setting (the more the songs are known, the more positively the songs are perceived). The songs in the virtual setting were already better known because they had also been sung in the in-person-sessions. Previous studies suggested that familiarity and positive value of music is an important determinant for positive effect on mood and well-being (Blood and Zatorre, 2001; Brown et al., 2004; Grebosz-Haring and Thun-Hohenstein, 2018).

We found that mood increased positively after choir singing, regardless of whether it was in-person or virtual setting. Furthermore, we observed that there was a pre-post decrease in sCort levels in the in-person setting, but not in the virtual setting. In contrast, the pre-post values of sCort in the virtual singing setting increased slightly. The result from the in-person singing setting is in accordance with previous studies in adults (Beck et al., 2000; Fancourt et al., 2016; Schladt et al., 2017) and children and adolescents (Grebosz-Haring and Thun-Hohenstein, 2018), in which group singing was connected to lower sCort levels, suggesting that active group singing might have stress-reducing effects. In particular, our study suggests that a biological response is more visible after active in-person singing than after virtual singing. This could be related to the fact that the participants in the virtual setting could only hear themselves but not the other singers. Participants could see and have virtual social contact with each other, but they were not physically together in the same place. Their feeling of belonging to the group and the “choir experience” might have been reduced by this fact. Similarly, experiencing strong group dynamics in an in-person setting can also help to reduce stress (Olff et al., 2013; Tarr et al., 2014; Koelsch, 2018). Indeed, we observed that participants experienced greater social contact in the in-person singing setting; in contrast, in the virtual singing setting, fewer social contacts were reported. However, this result should be taken with caution because more participants took part in the in-person singing setting than in the virtual singing one. On the other hand, the low increase in sCort levels in the virtual singing setting in our study could be specifically related to the framework of the virtual singing sessions, including technical problems and other possible co-variates such as mood of the choir conductor. These factors should be included and examined as possible confounders in any analysis involving larger group sizes. However, due to the small group sizes in this study, this would not result in statistically meaningful results. Finally, since in-person singing is physically more demanding (this refers to the sedentary situation of not needing to leave the house to join in, or being seated in front of the screen during each singing activity) than virtual singing, it is possible that the differences in sCort levels in both groups could be attributed at least in part to the varying levels of physical activity, which can influence endocrine system responses (see e.g., Kreutz et al., 2004).

Surprisingly, across the sample and independent of the setting, we found higher values of sAA after the singing sessions. This might be due to the fact that singing can have an activating effect (moving the mouth alone such as while chewing stimulates the flow of saliva), which is reflected in higher ANS activity.

In keeping with the sCort data, we observed a greater reduction of subjective stress experience in the in-person singing setting compared to the virtual setting. Moreover, the in-person singing setting had a larger increase in the subjective measures of mood state, in the dimension calmness, whereas in the virtual setting the dimension calmness remained almost the same. These findings confirm results from previous studies, which reported a decrease in sCort levels accompanying the subjective reduction in anxiety and stress (Fancourt et al., 2016) as well as an increase in positive mood (Beck et al., 2000; Fancourt et al., 2016; Schladt et al., 2017) in response to in-person group singing in other populations. Interestingly, we could observe that in general in participants in the virtual setting the values (both pre and post sessions) of positive mood were higher and stress level lower compared to the in-person setting. This could be related to the fact that the singing activity was more familiar to the participants. On the other hand, the generally lower values of positive mood and higher values of stress level in the in-person setting could be related to the fact that in-person choir singing has built-in tension due to the environment (without reflection on the choirmaster). Moreover, during the virtual setting, they were in their own environment.

The total score of the adaptive emotion regulation strategies for the feelings anger, anxiety and grief increased from the first to the second time period, which means that participants were able to regulate their emotions better. Interestingly, values decreased after the second time period, which started after the first lockdown and ended after the last virtual session. The study by Daffern et al. (2021) observes that for some participants, a virtual choir had a negative impact on their well-being, as it made them aware of what they really missed: being together. Not knowing when they will meet again makes people sad. This could be an explanation of our results. The total score of the maladaptive emotion regulation strategies decreased over the time periods. It is assumed that the emotion regulation strategies were used in a positive way and improved throughout the time periods. However, there may have been other influences on the data from this cross-sectional questionnaire besides the singing intervention, such as developmental changes and the lockdown situation, e.g. homeschooling.

We found that contingency of self-worth increased notably by the end of the study, whereas there was no change in self-worth and self-stability. Moreover, we observed that quality of life increased after the in-person choir sessions and stayed very high until the end of the virtual sessions. During the baseline measurement, the emotional functioning scale, which included questions related to anxiety, worry, sadness, anger, and insomnia showed the lowest value compared to the other scales. At the end of the virtual choir, an increase of values in all subscales (emotional functioning, school functioning, physical functioning, and social functioning) was observed. Previous studies showed the possibility that singing can improve quality of life in this group of participants (Grebosz-Haring and Thun-Hohenstein, 2018; Glew et al., 2020). The present data underlines previous findings. It is possible that because participants sang alone in the virtual setting, social demands decreased and thus contingent self-esteem, i.e., the comparison of oneself with internal and external values and norms, improved.

Finally, we observed a decreasing tendency in hair cortisol concentrations over the three time points. However, due to the small group size, the results should be considered with caution. Seasonal fluctuations could also have affected certain outcomes like saliva (Miller et al., 2016) and blood (Hadlow et al., 2014; Tendler et al., 2021) cortisol, because the hypothalamus-pituitary-adrenal axis varies with the season, although it is not empirically clear how strongly these influences are reflected in hair cortisol in humans (see Maimon et al., 2020). Previous findings are inconsistent and diverse, suggesting predominantly low hair cortisol in winter and high hair cortisol in summer (e.g., Braig et al., 2015; Fischer et al., 2017) but also high hair cortisol in winter (e.g., Abell et al., 2016) or no seasonal effect at all (e.g., van den Heuvel et al., 2020). Furthermore, hair cortisol itself (as well as other psychological outcomes) can also be affected by changed life circumstances and behaviors in the context of lockdown restrictions, such as less physical movement or different sleep patterns. Therefore, the results must be interpreted with caution.

### Strengths and Limitations

This study is one of the few studies examining both psychological and biological markers associated with choral singing in adolescents, over a longer time period, in both in-person and virtual settings. This provides a very important foundation for future studies that examine the health-promoting effects of in-person and/or virtual choir singing. With more technical possibilities, it may be possible at some point to improve virtual choir experiences and thus maximize the psychobiological effects, especially in possible future pandemic or crisis situations that involve social isolation. Furthermore, we managed to transform the in-person choir sessions into virtual sessions and thus succeeded in adapting to the situation so that we could continue our investigation and compare in-person and virtual singing within a slightly modified context in the time of the COVID-19-outbreak. An additional positive factor was that choir members had the possibility to have aesthetic experiences and maintain social contacts during the difficult time of isolation within the first lockdown.

Our study has a number of limitations. First, it has a small sample size, so the preliminary findings must be interpreted with caution. The small number of participants was related to the challenge in recruiting study participants, since some potential candidates showed interest, but few agreed to participate. One reason given for not participating was the time commitment involved, since many children already had several afternoon activities. The small number of participants and high drop-out rate should also be mentioned as a potential bias in the results because of the personality type or inclination to respond to the activity. It could be possible that those participants who persevered with the data collection and attendance were particularly responsive to singing as a beneficial activity, while those who dropped out were not. Perhaps the differences in both groups should be examined. However, due to the small group sizes, this would not result in meaningful results and without a larger sample it is of course difficult to investigate such theories. Another limitation is that the psychological baseline data were obtained at three time points: at the beginning of the in-person choir singing activity, and at the beginning and end of the virtual choir singing activity. Due to the lockdown, we were unable to obtain any data at the end of the in-person choir singing sessions. Therefore, the results from the first to the second time point (across time periods) should be viewed with care. Any changes in these measurements could be very strongly influenced by COVID regulations and cannot really be traced back to the choir intervention, since there is also no comparison or control group in the study design. The multitude of outcome measures, although they reflect a detailed picture of the effects of singing activities, also represent a limitation. Some data of the outcome measures were missing (e.g., CSIK) because participants were unwilling to provide them. The challenges in recruitment, including the small number of participants in our study and the number who withdrew, could be the result of an excess of questionnaires. As such, a future study should reduce the number of assessments and simplify the assessments that are carried out repeatedly. Moreover, due to the length of the study and the different seasons in which assessments were carried out (Assessment 1: autumn/winter, Assessment 2: spring, Assessment 3: spring/summer), we cannot exclude seasonal effects, rather than the effects of a specific musical activity, on certain outcomes like hair cortisol. A future study design should take this point into account. In our study, we controlled for liking of the music pieces and the choir sessions. Notably, our study did not include confounders such as the level of difficulty of the music, the singing condition of the participants, or the mood and engagement of the conductor. It is possible that biological markers such as cortisol are influenced by the conditions of the setting, rather than the in-person singing activity itself. However, due to the small number of participants, these conditions would not result in statistically meaningful outcomes. These conditions should be clearly included and investigated as possible confounders in a larger study.

Beyond these limitations, the COVID-19 pandemic presented major challenges to carrying out the study using conventional methodology. These challenges were due to the limitations in the interaction between participants and research assistants. Consequently, the research staff were not present when the saliva and hair samples were taken, so the procedure (despite detailed instructions) could not be monitored. However, there is research that shows that lay individuals can also carry out saliva and hair samples correctly if properly instructed (Schlotz, 2019; Skoluda et al., 2021). Moreover, storage conditions for the saliva samples between the coronavirus waves and from participant to participant were not standardized and may differ.

Notably, there were obstacles to virtual participation due to sound problems and a lack of reliable internet connections. It remains unclear whether these problems distracted and irritated the participants and whether they could lessen the positive effects of virtual singing sessions on cortisol levels.

Our virtual choir was a pilot project with preliminary findings that originated in the special circumstances surrounding COVID-19 and the resulting lockdown. Although there were recruitment challenges, the pilot study has shown that it is possible to conduct a study using group singing activities in children and adolescents. Further research with correspondingly large participant numbers is needed to explain the responses to virtual singing and the differences between in-person and virtual conditions. Singing is suggested to benefit psychological and biological outcomes in children and adolescents. However, despite our current knowledge, we must better understand the psychobiological mechanisms underlying choral singing. A larger study with a control group is recommended to investigate this issue in this population.

## Data Availability Statement

The raw data supporting the conclusions of this article will be made available by the authors, without undue reservation.

## Ethics Statement

The study involving human participants was reviewed and approved by Salzburg State Ethics Committee. Written informed consent to participate in this study was provided by each participant and participants’ legal guardian/next of kin.

## Author Contributions

KG-H and LT-H conceived and designed the study. UMN and ACF contributed to the study design. KG-H and AKS-W performed the collection of data. SS performed the statistical analysis. KG-H and AKS-W contributed to the data analysis. The biochemical analyses were performed under the supervision of NS and UMN. KG-H drafted the first version of the manuscript. KG-H and AKS-W wrote sections of the manuscript. LT-H, UMN, ACF, and NS were involved in the final drafting of the manuscript, and provided critical feedback on the basis of their special areas of interest, which were incorporated into the final draft of the manuscript. All authors approved the submitted version.

## Conflict of Interest

The authors declare that the research was conducted in the absence of any commercial or financial relationships that could be construed as a potential conflict of interest.

## Publisher’s Note

All claims expressed in this article are solely those of the authors and do not necessarily represent those of their affiliated organizations, or those of the publisher, the editors and the reviewers. Any product that may be evaluated in this article, or claim that may be made by its manufacturer, is not guaranteed or endorsed by the publisher.

## Footnotes

1
http://www.clinicaltrials.gov/ct2/show/NCT04454736?cond=singing&draw=3&rank=14

## Appendix

### Appendix 1

Songs used in the choir sessions.

(1) “Let It Be” (written by Lennon/McCartney, performed by “The Beatles” and released in 1970).
(2) “Count on me” (written by Bruno Mars, Philip Lawrence, and Ari Levine, performed by Bruno Mars and released in 2011).
(3) “You raise me up” (music by Rolf L vland, lyrics by Brendan Graham, released in 2002).
(4) “Das Maultier und das Faultier” (music by Uli F hre, lyrics by J rg Ehni).
(5) “99 Luftballons” (composed by Uwe Fahrenkrog-Petersen, German lyrics by Carlo Karges, English lyrics by Kevin McAlea, released in 1983 in West Germany and 1984 in the United Kingdom) (introduced in the virtual choir sessions).
(6) “Zombie” (written by Dolores O’Riordan, performed by The Cranberries and released in 1994) (introduced in the virtual choir sessions).
(7) “Sweet Dreams” (written by Annie Lennox and David A. Stewart, performed by Eurythmics and released in 1983) (introduced in the virtual choir sessions).
(8) “Poco a poco” (Arr. Andy Icochea Icochea, Rodolfo C zares, abducted in Mexico on July 9, 2011).
(9) “Jikijela” (transcription by Elizabeth Oltedal).

### Appendix 2

Outline of the singing session.

The following goals are established:

*Musical development*

- Learning to use the singing voice in a healthy and sonorous manner.
- Getting to know and to play songs in different styles and languages.
- Singing well-known and popular songs.
- Acquiring basic skills for musical understanding (score, rhythm, and pitch).
- Homophony and polyphony, canon.

*Personality development*

- Being aware of one’s voice (self-awareness).
- Learning to listen to each other (musically as well as interpersonally).
- Expressiveness – expressing the content of the voice.
- Strengthening group cohesion through common goals (and hopefully, success stories).
- Learning to maintain a self-, body-, and effect-conscious appearance.

**Appendix Table:** The breakdown of each lesson (45 min) is shown in the following table.

Warm-up.	Physical activation, vocal warm-up (sounding, making the voice sound, coming out of the speaking voice, and raising high and low resonances), attention exercises.	10′
Sing a well-known song (rehearse if necessary).	Passion for singing is awakened.	5′
Musical work on new or previously learned songs.	Varied, multifaceted rehearsals of musical elements: fast / slow; rhythmic / sustained; sonorous / voice-oriented; and active / passive participation in the rehearsal (e.g., in a voice rehearsal).	15′
Play a game to release tension.	Preferably includes personality-developing content.	7′
Qualitative singing of a previously learned song.	Define the qualitative claims. Consciously demand a self-confident appearance and awareness of the voice.	4′
End.	Energizing ending with a final song.	4′
